# Molecular Computing and Bioinformatics

**DOI:** 10.3390/molecules24132358

**Published:** 2019-06-26

**Authors:** Xin Liang, Wen Zhu, Zhibin Lv, Quan Zou

**Affiliations:** 1 School of Mathematics and Statistics, Hainan Normal University, Haikou 570100, China; 2 Institute of Fundamental and Frontier Sciences, University of Electronic Science and Technology of China, Chengdu 610054, China; 3 Center for Informational Biology, University of Electronic Science and Technology of China, Chengdu 611731, China

**Keywords:** molecular computing, bioinformatics, machine learning, protein, DNA, RNA, drug, bio-inspired

## Abstract

Molecular computing and bioinformatics are two important interdisciplinary sciences that study molecules and computers. Molecular computing is a branch of computing that uses DNA, biochemistry, and molecular biology hardware, instead of traditional silicon-based computer technologies. Research and development in this area concerns theory, experiments, and applications of molecular computing. The core advantage of molecular computing is its potential to pack vastly more circuitry onto a microchip than silicon will ever be capable of—and to do it cheaply. Molecules are only a few nanometers in size, making it possible to manufacture chips that contain billions—even trillions—of switches and components. To develop molecular computers, computer scientists must draw on expertise in subjects not usually associated with their field, including organic chemistry, molecular biology, bioengineering, and smart materials. Bioinformatics works on the contrary; bioinformatics researchers develop novel algorithms or software tools for computing or predicting the molecular structure or function. Molecular computing and bioinformatics pay attention to the same object, and have close relationships, but work toward different orientations.

## 1. Introduction

The origin of molecular computing was as early as 1961, which was conceived by Feynman [1]. Due to the limitations of experimental conditions, materials, and biotechnology at that time, Feynman’s idea was not really realized. In the following decades, biological theories have been evolving, and new biotechnology and experimental methods have been constantly emerging, which paved the way for the final reality for molecular computing. In 1994, Adleman [1] put forward a DNA molecular biological calculation method based on the Hamilton graph and successfully achieved molecular computing in DNA solution for the first time. Adleman’s pioneering work opened a new field for computational science, which was of great significance and soon gained extensive attention from researchers in the field of mathematics, computer, biology, etc. In addition, other biological computing models, such as membrane computing [2], bacterial computing [3], evolutionary calculation [4,5], and virus calculation [6] have been proposed and implemented.

With the development of new generation sequencing technology, the scale of DNA, RNA, and protein biological database has been increasing dramatically [7]. An era of biological big data set in. How to efficiently analyze biological big data becomes a great challenge. Bioinformatics is an important means to cope with this challenge [8,9]. Bioinformatics combines the tools of mathematics, computer science, and biology to more efficiently elucidate and understand the biological implications and significance for a variety of sequence and structure data as well as other biological data, which has enormously promoted the research and development of many areas relative to biology. For instance, specific biological macromolecules identification and functional analysis could be achieved via bioinformatics [10,11]. By means of bioinformatics, we could uncover the relationship between genes and diseases and analyze the mechanism of diseases, both of which would benefit diseases diagnosis, diseases treatment, and even epidemic prevention [12,13]. Using the relationship between the structure and function of biomolecules gained by bioinformatics, we could analyze the effective composition of complex drugs, discover the target of new drugs, and design new drugs [14]. All of these achievements come with new software, new algorithms, and new tools originated from continuously evolving bioinformatics.

After a rigorous review process, 25 papers submitted from numerous countries including China, Malaysia, South Korea, Poland, Saudi Arabia, and so on are published in the special issue. Twenty-two of these papers are directly related to topics of molecular computation and bioinformatics. Three of them are new areas with overlapping frontiers, which are assigned to bio-inspired research areas. It is hoped that the researchers’ results and perspectives in the issue will arouse readers’ interest and inspire readers.

## 2. Molecular Computing

Differing from traditional silicon-based computing, DNA computing is an integrated technology with DNA molecules, biochemical reactions, and molecular biology. As the field has gained insight into the molecular structures, physical–chemical properties and biomechanisms of DNA, DNA computing has been developing rapidly and become an increasingly important branch in the field of computing. The DNA double strands complementary hybridization rule is the cornerstone for DNA computing. Based on this, it uses well-designed DNA sequences with a variety of carefully selected parameters such as the position binding force of the double-strand formation to realize the chemical reaction of the DNA chain system for DNA computing. Two articles in the issue focus on DNA computing. Han et al. [15] designed an 8-bit adder/subtractor with domain tags based on DNA chain displacement. The adder/subtractor used different domains to represent 0 and 1 signals instead of high and low DNA concentration. Their simulation results proved the feasibility and accuracy of the adder/subtractor logic calculation model based on the domain label, which could extend its application for molecular logic circuits. Beak et al. [16] developed an enzyme weight-updating algorithm on the basics of DNA molecular learning for future smart molecular computing systems. The new algorithm used a hypernetwork model, which integrated the internal circulation structure of DNA and ensemble learning to update the enzyme weight. It enabled the enzyme to be used for the large-scale parallel processing of DNA. At the same time, the intuitive method of DNA data construction in Beak’s work could significantly reduce the number of unique DNA sequences that are needed for covering the large search space of the feature set. It was an algorithm that realized the combination of molecular computation and machine learning.

Along with DNA computing as one of the biological computing models, there are other forms of biological computing, including membrane calculation [17,18,19], evolutionary calculation [4,5], virus calculation [6], etc. The purpose of bacterial computing is to build “bacterial computers” to solve complex problems. In this issue, Wang et al. [20] proposed a bacterial and plasmid computing system (BP system). Two bacteria, 34 plasmids, and two genes were used to build two BP systems to demonstrate the possibility of building powerful bacterial computers.

## 3. Bioinformatics

### 3.1. Biomolecules Structure and Function Analysis

The analysis of the structure and function of biomolecules is an important area in biology, which involves multiple subjects such as protein secondary structures, protein and gene identification, and the analysis of specific functional binding sites for DNA and proteins, etc. The algorithm tools and software provided by bioinformatics greatly advance the progress in these fields. This special issue contains six related papers to the subtopic. Ping et al. [21] utilized bioinformatics tools and software such as the Basic Local Alignment Search Tool (BLAST), MEGA7.0, GSDS2.0 etc. to identify laccase gene families from three different Brassics. A series of changes under the stress for BnLACs (laccase genes from the Brassica napus genome) expression was investigated by RNA sequencing and quantitative real-time polymerase chain reaction and resulted in better insights for BnLACs’ evolutions and functions. Su et.al. [22] used TransportTP, WOLF-PSORT, MEME, and other bioinformatics tools to conduct genome-wide identification and comparison of oligopeptide transporter (OPT) family genes for ginseng and 11 flowering plants. They also analyzed the expression, evolution, and biological function of OPT family genes. Their work improved the interpretation of metabolic transport mechanism and signal transduction during the cultivation of ginseng plants. Miskiewicz et al. [23] applied WebLogo, ContextFold, RNApdbee, RNAComposer and other tools to discover structural motifs in miRNA precursors from the Viridiplantae kingdom, and they revealed the secondary structural pattern of microRNA. Kalidasan et al. [24] studied the iron harvesting system of stenotrophomonas maltophilia using BLAST tools and biological experimental techniques, and proved that stenotrophomonas maltophilia acquired iron during iron starvation and used specific iron sources. Zhang et al. [25] proposed a method called Reprsent Concat, which integrated multiple heterogeneous interactive networks. The method was able to infer gene function. More heterogeneous network methods and applications could be referred to the review [26]. Feng et al. [27] carried out a support vector machine ensemble classifier algorithm to construct a recognition method for D modification site in the saccharomyces cerevisiae transcriptome. They achieved an accuracy of 83.09% with a Matthew correlation coefficient of 0.62. Using machine learning to predict modification sites is currently a hot topic in the field of biological information. Some state-in-art deep learning methods have been developed for predicting N6-methyladenosine(m6A) [28], N4 -methylcytosine (4mC) [29], and so on. 

In addition, molecular topological index is defined as the invariant of the distance or degree of the vertex in the molecule, which is used to describe molecules and is useful for predicting the physical and chemical properties of proteins, DNA, and RNA and for verifying macromolecular structural characteristics. In the issue, Zhang et al. [30] employed two classical operations in graph theory, i.e., Cartesian product and graph connection, to construct an edge version topological index for atomic bond connection and geometric frameworks. They gave the proof detail of theory involved.

### 3.2. Drug Research and Development (R&D) 

It is well known that drug R&D is notoriously long and expensive. A study published in Nature Medicine in 2010 found that a drug took an average of 13 years and cost $1.8 billion to develop from its initial laboratory study to its final release [31]. However, bioinformatics enables us to effectively reduce the drug R&D period and expense, thus making it more productive for drug R&D. In the issue, Chen et al. [32] gave a comprehensive overview of machine learning algorithms for drug-target interaction prediction, and also summarized a brief list of frequently used databases. They introduced the principles, pros, and cons of representative methods, especially the latest new algorithms, and expounded the challenges and future trends for drug–target interaction prediction. In response to the challenge regarding the dense protein interaction network identification algorithm not being suitable for sparse protein–protein interaction (PPI) networks, Cao et al. [33] developed a new method for identifying punitive protein complexes based on penalized matrix decomposition (PMD). This method surpassed previously reported methods, and achieved an ideal overall f-measure performance, better accuracy (ACC), and a maximum matching rate. Chen et al. [34] constructed a prediction algorithm for the outflow mechanism of p-glycoprotein compound substrates, which could be used for drug discovery and development. In Chen’s work, a new hierarchical support vector regression scheme was built to study the nonlinear quantitative structure–activity relationship (QSAR) and explore the complex relationship between descriptor and outflow rate. With deep learning framework, Hu et al. [35] proposed a general method (SDHINE) for predicting adverse drug reactions by embedding heterogeneous networks, which integrated protein–protein interaction (PPI) information into drug embedding. Indeed, machine learning—including deep learning—is so helpful for drug R&D that quite a mass of works has published in recent years. For example, besides in this issue, Su et al. [36] used different deep learning methods to predict the efficacy and adverse reactions of cancer drugs. Ding et al. predicted the correlation between drug targets [37,38] and drug side effects [39,40] with types of machine learning methods.

Additionally, a review of the use of bioinformatics to identify Chinese herbs is presented in this special issue. Han et al. [41] outlined the two kinds of technology—biochip and DNA barcode—and their application for the identification of Chinese herbal medicine. Chinese herbs generally came from a wide range of sources, and some of them seemed to be so similar that it was hard to distinguish them by shape, color, or other apparent characteristic. However, with bioinformatics strategic methods, the identification of Chinese herbal medicine composition was speedy and accurate, as mentioned by Han et al.

### 3.3. Disease Analysis and Research

Bioinformatics affords us a feasible and novel means for studying on diseases diagnosis, treatment, and even on transmission mechanism. This special issue includes several related papers. Oh et al. [42] used the TRANSFAC tool and biological experimental technique to study the therapeutic effect of the HIF-1 alpha hypoxia inducer on peri-implant bone formation in diabetic mice, and concluded that the local application of HIF-1 alpha induced gene expression and growth promotion of the bone around the implant. On the basis of amino acid mutation, Qiang et al. [43] established a prediction model of avian influenza transmission from bird to human via using random forest, support vector, and other machine learning methods. Their research concluded that there were three molecular patterns of avian-to-human transmission for avian influenza that existed in nature. Xu et al. [44] exploited a support vector machine (SVM) to discriminate genes of Alzheimer’s syndrome (AD) with an accuracy of 85.7%. Zakariah et al. [45] used the new generation sequencing technology, Hum-mPLoc 3.0, and other tools to study the human mycoplasma protein targeting the endoplasmic reticulum and its effect on the causes of prostate cancer. Their prediction found that intercellular infection in host cells was capable of leading to prostate cancer. Abnormal miRNA expression in various environmental factors (such as anxiety, alcoholism, etc.) gives rise to a series of diseases. The identification of the relationship between miRNA and environmental factors would facilitate the curing of diseases. Luo et al. [46] developed a new algorithm that integrated multiple types of biological information to reveal the interaction between miRNA and environmental factors, and the area under curve(AUC) of the algorithm reached 0.8208. Similarly, web-based methods have also been applied to predict the relationship between miRNA and disease [47,48,49,50]. The gene fusion structure is a common somatic mutation in cancer genome. The identification of drivers for fusion structures is of great importance for many downstream analyses, and is useful for clinical practice. Xu et al. [51] proposed a new algorithm for the stable identification of fusion structure driver genes. The algorithm took the gene network as a priori information and estimated the driver gene according to the destructive hypothesis.

Beyond the above-mentioned studies, this issue collectsan article on large-scale biomedical text data mining. Xing et al. [52] developed a parallel processing framework called ParaBTM for biomedical text mining on supercomputers. When running on the Tianhe-2 supercomputer, it took less than 12 h to process 60178 PubMed full texts by ParaBTM.

## 4. Bio-Inspired Research

The remaining three papers are on cross-cutting research and organized as a bio-inspired research area. Inspired by DNA sequences with the biological properties such as parallel computation and low energy consumption, DNA computation and DNA coding are widely used in image encryption [53]. In this issue, Wang et al. [54] introduced their new algorithm for correcting image encryption errors by using DNA coding. Hamming distance was used to reduce the similarity of DNA sequences for error correcting. Image edge detection is a fundamental task in image processing and computer vision. Yuan et al. [55] applied the enzymatic numerical P system (ENPS) to solve image edge detection problems. ENPS was a cell-like P system with a nested membrane structure consisting of four membranes. The calculation of edge detection was carried out in parallel among the three inner membranes. Exploring and examining the causal relationship between variables has shown great practical value in recent years, and could be used for scientific discovery from big data. Hong et al. [43] constructed the so-called K2 and BSO combined causal discovery optimization algorithm, which mimicked the human way of solving problems with brainstorming. Their algorithm took advantage of the K2 mechanism and used BSO to design the optimal topological order of searching nodes instead of the traditional graph space, which was able to solve the problem that the traditional algorithm could not work properly, since the graph space was too large.

## 5. Conclusions

This special issue covers several emerging topics in the fields of molecular computing and bioinformatics, which is supposed to intrigue a wide variety of readers. It must express gratitude to the Molecules editorial board for offering such a good opportunity to organize such a special issue. It must also appreciate the efforts of the reviewers to ensure the high quality of this special issue. Finally, it is thankful for all those who have contributed to this issue. More authors and readers are expected to contribute to Molecules in the future.
